# DSCAM-AS1 promotes the development of prostate cancer

**DOI:** 10.1007/s12672-024-00931-3

**Published:** 2024-04-11

**Authors:** Lin Cheng, Shuhui Li, Deqi Jiang, Jianchao Zhang

**Affiliations:** 1 Department of Urology Surgery, Shandong Provincial Third Hospital, No. 12, Wuyingshan Middle Road, Tianqiao District, Jinan, 250031 Shandong China; 2 Department of Joint Surgery, Shandong Provincial Third Hospital, Jinan, 250031 Shandong China

**Keywords:** Prostate cancer, DSCAM-AS1, miR-338-3p, Prognosis, Cellular processes

## Abstract

**Purpose:**

The purpose of this study was to investigate the role of lncRNA DSCAM-AS1 in prostate cancer to find new therapeutic targets and promote the research progress of prostate cancer.

**Methods:**

RT-qPCR was used to detect DSCAM-AS1 expression in prostate cancer tissues, normal tissues, human normal prostate epithelial cells (RWPE), and four prostate cancer cell lines. The clinical and prognostic role of DSCAM-AS1 was evaluated by the Kaplan–Meier curve and chi-square test. Secondly, a dual luciferase reporter gene assay was used to study the regulatory mechanism between miR-338-3p and DSCAM-AS1. Finally, the roles of DSCAM-AS1 and miR-338-3p in prostate cancer cell proliferation and metastasis were explored by CCK-8 and Transwell assays.

**Results:**

It was found that DSCAM-AS1 upregulation could serve as a warning of deterioration and poor prognosis in prostate cancer patients, and that knockdown of DSCAM-AS1 expression inhibited the progression of prostate cancer cells. In addition, miR-338-3p, a target of DSCAM-AS1, was found to be down-regulated in prostate cancer cells and miR-338-3p knockdown could reverse the inhibitory effect of DSCAM-AS1 silencing on prostate cancer.

**Conclusion:**

DSCAM-AS1 is up-regulated in prostate cancer and regulates the progression of prostate cancer cells by targeting miR-338-3p.

**Supplementary Information:**

The online version contains supplementary material available at 10.1007/s12672-024-00931-3.

## Introduction

Prostate cancer is one of the highest morbidity and fatality rates among male malignant tumors [1]. Prostate cancer is difficult to diagnose early, which has low antigen specificity and strong stealthie. It has a poor prognosis due to strong invasiveness, high postoperative recurrence rate, and metastasis rate [2, 3]. Therefore, enhancing early diagnosis can significantly raise the survival rate of individuals with prostate cancer. Previously, the diagnosis of prostate cancer lacked biomarkers with high sensitivity and specificity, therefore, it is critical to find a marker to compensate for the absence through molecular biology and pathology investigations.

Long non-coding RNA (lncRNA) is becoming more significant in the regulation of the occurrence and growth of cancer tumors as research advances [4, 5]. With the in-depth study of LncRNA and the pathogenesis of prostate cancer, more and more evidence shows that LncRNA is involved in the occurrence and development, drug resistance, prognosis evaluation, and other processes of prostate cancer, and has the potential to be used as a therapeutic target [6]. SNHG1 can activate the EMT (Epithelial Mesenchymal Transformation) pathway to promote the malignant progression of prostate cancer and is expected to be a therapeutic target for prostate cancer [7]. The lncRNA CCAT1 can deteriorate prostate cancer to denervation-resistant prostate cancer by binding to intracellular miR-28-5p, thereby altering androgen receptor co-activators and affecting the AR signaling pathway [8]. It has been shown that aberrant expression of the lncRNA SChLAP1 is associated with the development of lethal prostate cancer, whereas high expression levels of SChLAP1 in prostate cancer tissues are significantly associated with biochemical recurrence, clinical progression, and mortality [9]. In addition, SChLAP1 is readily detectable in urine, which adds potential applicability for guiding therapy [10]. Therefore, the study of lncRNA in prostate cancer can improve our knowledge of prostate cancer and thus improve its treatment.

Through the GEO database (https://www.ncbi.nlm.nih.gov/gds), data sets GSE179321, GSE115414, and GSE229904 were used in this investigation to seek differentially expressed lncRNAs, and C1QTNF3-AMACR, DSCAM-AS1, and SCHLAP1 were identified. In tumor and genome-wide research related to cancer, C1QTNF3-AMACR has not been reported. It was reported that SCHLAP1 is associated with the development of prostate cancer, according to various articles. For example, Huang et al. discovered that SCHLAP1 can promote the development of the prostate by mediating miRNA [9]. However, it has not been reported that the role of DSCAM-AS1 in prostate cancer and molecular regulation mechanisms. According to Mehra et al. upregulation of SCHLAP1 can signal a bad prognosis for prostate cancer [11]. DSCAM-AS1 is reported as an important therapeutic target in cancer [12], which has been discovered in abnormal expression in pancreatic cancer [13], breast cancer [14], and endometrial cancer [15]. Therefore, we believe that DSCAM-AS1 has some research reference value and explore its role in prostate cancer for the first time.

To find new therapeutic targets for prostate cancer and improve early diagnosis and prognosis, we collected clinical trial data of patients and conducted a series of related experiments to reveal the clinical significance and biological role of DSCAM-AS1 in prostate cancer.

## Materials and methods

### Clinical data and tissue

This study obtained clinical data and tissue samples from 113 prostate cancer patients who did not undergo any anti-tumor therapy other than surgical resection in Shandong Provincial Third Hospital during 2016–2017. This study had also been approved and executed by the Shandong Provincial Third Hospital Ethics Committee. Each patient consented voluntarily and in full knowledge of the study’s purpose. Through telephone follow-up and regular reviews, the patient’s postoperative status was also monitored and documented for five years. The matched tumor and normal tissue samples have been verified by at least two pathologists, andwere stored at − 80 °C.

### Selection of lncRNA and miRNA

The GEO Datasets are filtered by the keyword "prostate cancer", selecting "Series" and "Homo sapiens". Three datasets, GSE179321, GSE115414 and GSE229904, were selected (Additional file 1: Table S1). The three datasets were analyzed using GEO2R, and the results were derived, missing items and duplications were removed, thresholds were set to *p*-value < 0.05 and |LogFC| > 2, and three groups of DEGs were finally screened. Using Starbase (https://rnasysu.com/encori/index.php), lncRNASNP (10.1093/nar/gkac981) and lncBook (https://ngdc.cncb.ac.cn/lncbook/home) to predict the downstream miRNAs of DSCAM-AS1 in prostate cancer and download dataset. Using online Venn diagram software (https://www.bioinformatics.com.cn/static/others/jvenn/example.html) map the intersection of three groups of DEGs and downstream miRNAs, separately.

### Cell culture and cell transfection

A human normal prostate epithelial cell (RWPE) and four prostate cancer cells, including LNCap, PC-3, DU145, and 22Rv1 were used for the experiment, which were obtained from the Shanghai Cell Bank (Shanghai, China). In DMEM, cells were cultured and supplied with 10% FBS and 0.1% penicillin–streptomycin (Gibco, USA), and the culture environment was maintained at 37 °C and 5% CO_2_. According to the Transfection Kit directions, si-DSCAM-AS1 was mixed with Lipo-fectamine 2000 (Invitrogen, USA), which was cultured in serum-free DMEM. miR-338-3p mimics, miR-338-3p inhibitors, and negative controls were transfected into LNCap and DU145 (random selection). After 6 h of culture, transfected cells were obtained by replacing the substrate with a complete medium and continuing culture for 48 h.

### qRT-PCR

According to the gene serial number in GenBank, primers were designed with Premier 5.0 software: DSCAM-AS1 and miR-338-3p and corresponding reference gene GAPDH and U6 and sent to Shanghai Bioengineering Company for synthesis (Additional file 2: Table S2). Firstly, using Trizol reagent (Invitrogen, USA), tissues and cells were lysed to extract total RNA. High quality samples with absorbance (OD260 nm/OD280 nm) values of 1.9–2.1 was selected by NanoDrop ND-1000 spectrophotometer (NanoDrop, USA) for follow-up experiments. RNA samples were reverse transcribed to cDNA by sequential addition of reagents according to the reverse transcription kit PrimeScript RT Enzyme Mix I kit (TaKaRa, Japan). According to the instructions of SYBR Green qPCR Super Mix (Invitrogen, USA), the system was prepared: 10 μL 2 × PCR Mixture, 10 μmol/L forward and reverse primer 0.4 μL each, 1 μL template cDNA, 8.2 μL ddH_2_O. The above systems were detected by CFX96 fluorescence quantitative PCR instrument (ABI, USA) with the cycle parameters of 95 ℃ for 5 min. 95 ℃ 10 s, 59 ℃ 20 s, 72 ℃ 34 s, a total of 40 cycles. The relative expression is calculated by the 2^−ΔΔct^ method.

### Dual luciferase reporter assay

Construct the vectors of DSCAM-AS1 wild-type (WT-DSCAM-AS1, with predicted binding sites) and DSCAM-AS1 mutant (MT-DSCAM-AS1, with mutant sites), which were co-transfected with miR-338-3p mimics or miR-338-3p inhibitors into LNCap and DU145 cells by Lipofectamine 2000 (Invitrogen, USA). After 48 h, using a Dual Luciferase Reporter Assay System (Beyotime), the luciferase activities of DSCAM-AS1 were detected.

### CCK8 assay and transwell assay

Untransfected LNCap and DU145 normal cells were selected as the control group, and si-NC, Si-DSCA-AS1, si-miR-NC, and si-miR-inhibitor were transfected into LNCap and DU145 cells, respectively according to the transfection method in 2.3 for CCK8 assay and Transwell assay. When the CCK8 assay was performed, cells with different treatments were cultured on 96-well plates, and 10 μL of CCK8 solution (Dojindo, Japan) was added at 0, 24, 48, and 72 h, and the OD450 value was determined after 2 h by NanoDrop ND-1000 spectrophotometer (NanoDrop, USA).

When the Transwell assay was performed, after resuspending all the above cells in serum-free medium, the suspension of cells was inoculated in the upper layer of Transwell (EMD Millipore, USA) and the lower layer was treated with a high-serum medium. Three samples were replicated in each group and incubated in a 37 °C, 5% CO_2_ incubator for 48 h. Then they were washed, fixed, and finally stained with 0.1% crystal violet aqueous solution for 30 min. After the Transwell was inverted and let to dry, microscopy to observe the cells and record.

### Statistical analysis

Using SPSS 26.0 and GraphPad Prism 7.0, the data was analyzed. Student's t-test was used for two-group comparisons, while one-way ANOVA was used for multi-group comparisons. The relationship between DSCAM-AS1 and clinicopathological characteristics of prostate cancer patients was evaluated by chi-square test, and prognostic factors were assessed using multivariate Cox regression analysis, and the relationship between DSCAM-AS1 expression and patient survival time was examined using Kaplan–Meier log-rank tests. The expression for all data was as mean ± SD. The difference was shown to be statistically significant with *P* < 0.05.

## Results

### Expression and clinical significance of DSCAM-AS1 in prostate cancer

**Fig. 1:**
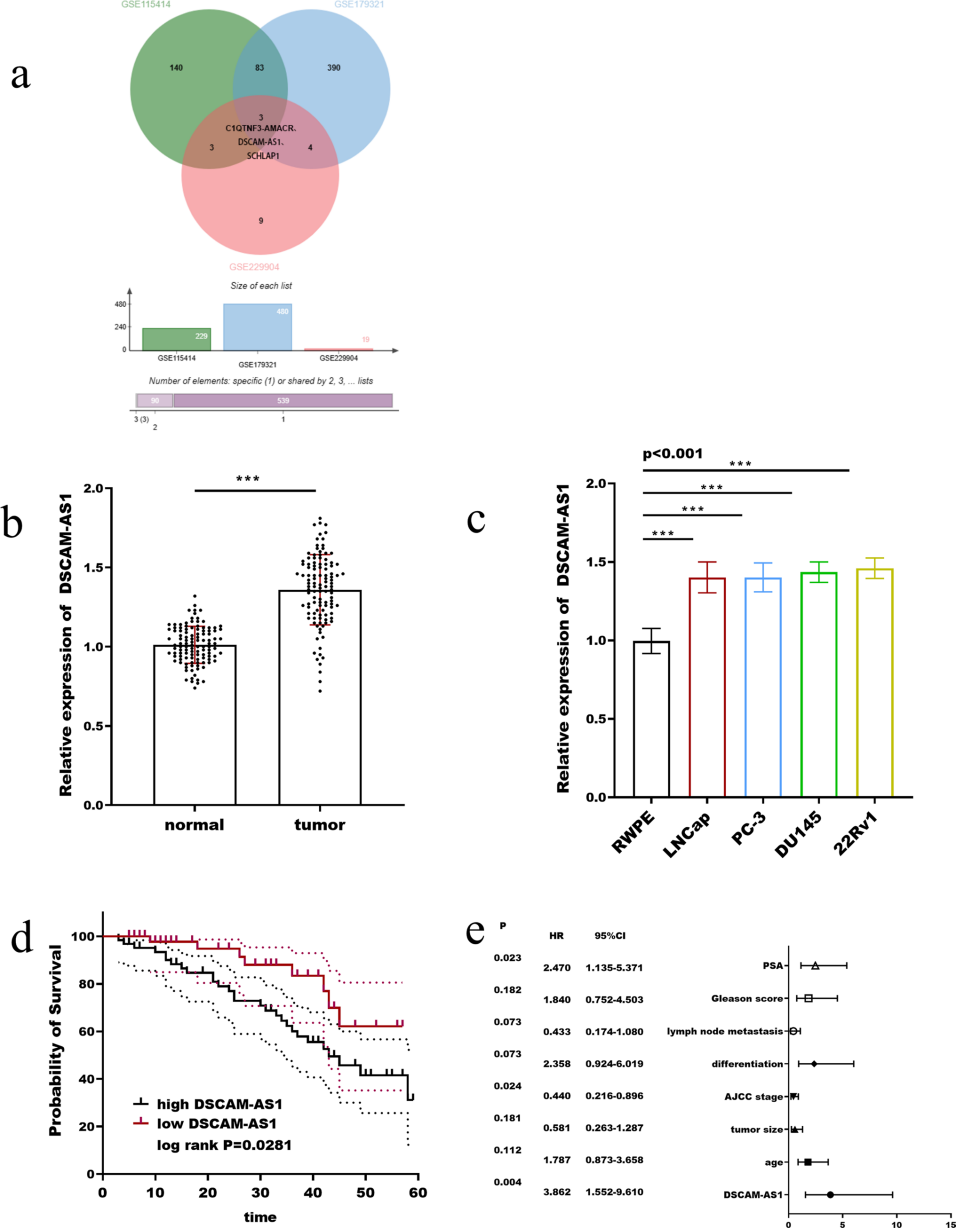
Expression and clinical significance of DSCAM-AS1 in prostate cancer. Screening for differentially expressed genes in the GSE179321, GSE115414, and GSE22990 datasets of the GEO database (**a**). DSCAM-AS1 was significant upregulated in prostate cancer tumor tissues compared with normal tissues (**b**) and cells (**c**). The higher the expression level of DSCAM-AS1, the worse the prognosis of prostate cancer patients (**d**). DSCAM-AS1, AJCC stage, differentiation, lymph node metastasis and PSA all have potential as prognostic markers for prostate cancer patients (**e**). ***p < 0.001, compared with normal tissues and normal cells

As shown in Fig. 1a, three differentially expressed genes were screened out according to the data sets GSE179321, GSE115414, and GSE229904, and through literature investigation, DSCAM-AS1 was finally identified as the target gene of this study. DSCAM-AS1 has been screened as a candidate biomarker for prostate cancer. DSCAM-AS1 was significantly upregulated in prostate cancer tissues (Fig. 1b) and cells (Fig. 1c) compared with normal tissues and cells. As shown in Table 1, according to the average expression of DSCA-AS1, 113 prostate cancer patients participating in the study were divided into the low-DSCA-AS1 and the high-DSCA-AS1, and clinical data of patients were statistically collected, which included DSCAM-AS1 expression, age, tumor size, AJCC stage, differentiation, lymph node metastasis, Gleason score, PSA, etc. The results showed that there was a significant difference in the clinical characteristics of patients in the high and low DSCAM-AS1 expression groups (*p* < 0.01), except for age (*p* = 0.269) and differentiation (*p* = 0.105). The survival rate of patients was significantly correlated with DSCSCAM-AS1 (log-rank *p* = 0.0281, Fig. 1d). The higher the expression level of DSCAM-AS1, the lower the 5-year survival rate and the worse the prognosis In addition, DSCAM-AS1, AJCC stage, differentiation, lymph node metastasis, and PSA all have potential as prognostic markers for prostate cancer patients (Fig. 1e).

**Table 1:** The association of DSCAM-AS1 with clinicopathological features of patients

Variant	Cases (n = 113)	lncRNA DSCAM-AS1 expression		*p*
		Low (n = 51)	High (n = 62)	
Age				0.269
< 60	53	21	32	
≥ 60	60	30	30	
Tumor size (cm)				0.028
≤ 4	58	32	26	
> 4	55	19	36	
AJCC stage				0.011
I–II	77	41	36	
III	36	10	26	
Differentiation				0.105
Well-moderate	80	40	40	
Poor	33	11	22	
Lymph node metastasis				0.026
Yes	39	12	27	
No	74	39	35	
Gleason score				0.022
≤ 7	76	40	36	
> 7	37	11	26	
PSA (ng/mL)				0.014
≤ 10	75	40	35	
> 10	38	11	27	

### The mechanisms of DSCAM-AS1in prostate cancer

**Fig. 2:**
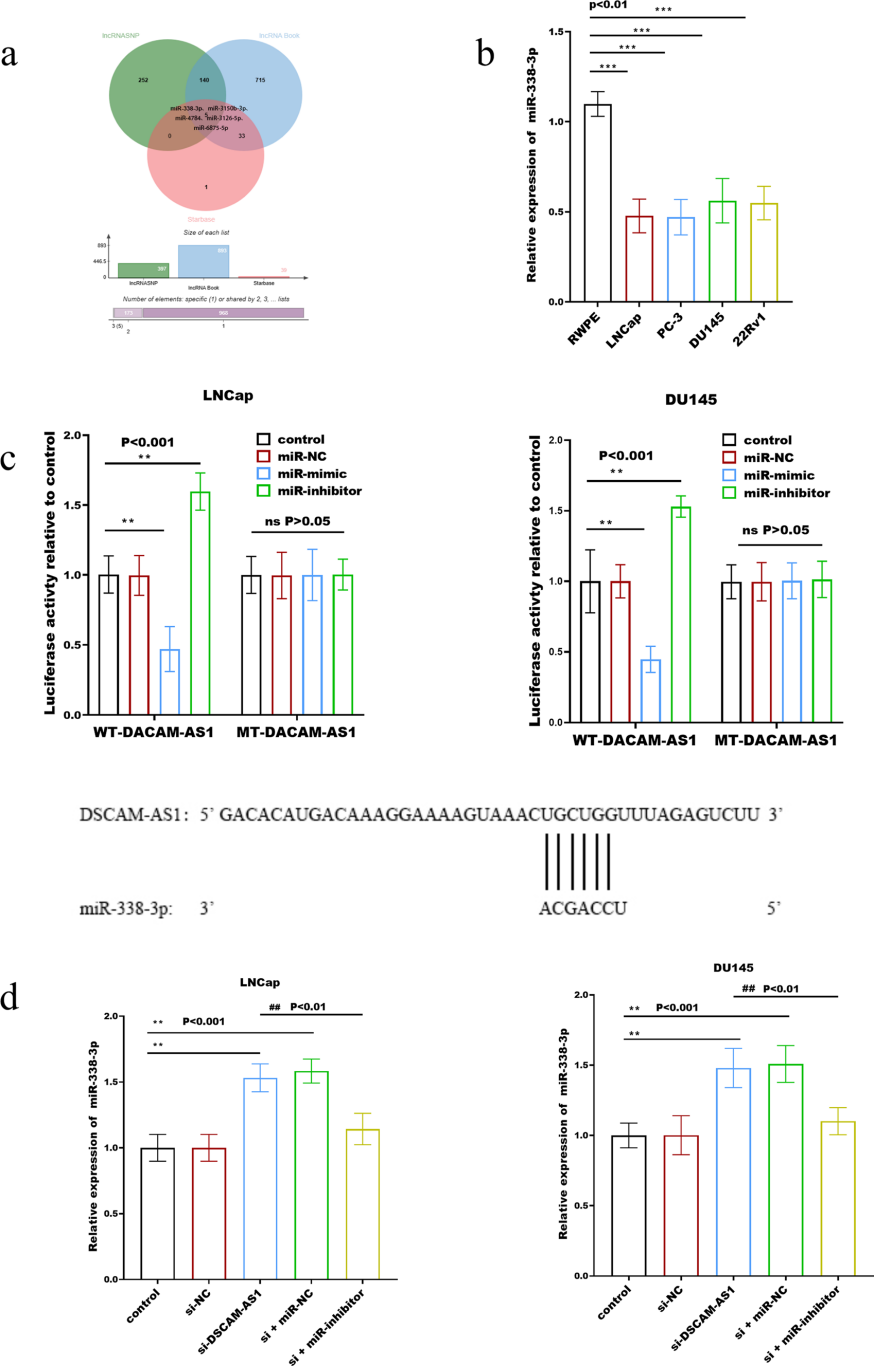
The mechanisms of DSCAM-AS1 in prostate cancer. The downstream miR-338-3p of DSCAM-AS1 was screened by database including lncRNASNP, lncRNA Book, and Starbase (**a**). Compared with normal cells, the expression of miR-338-3p in prostate cancer cells was significantly downregulated (**b**). The activity of DSCAM-AS1 luciferase dramatically decreased after transfection of miR-338-3p mimics, but it significantly enhanced after transfection of miR-338-3p inhibitors (**c**). miR-338-3p expression increased considerably following DSCA-AS1 knockdown, which was reversed by transfection with the miR-338-3p inhibitor (**d**). ***p < 0.001, **p < 0.01, compared with normal cells or control group, ##*p* < 0.01 compared with the si-DSCAM-AS1 group

The downstream miRNAs of DSCAM-AS1 were screened, and 397 downstream miRNAs were screened from lncRNASNP, 893 from lncRNA Book, and 39 from Starbase, which were intermixed to finally screen out 5 miRNAs (Fig. 2a). By using PCR, it was demonstrated that only miR-338-3p was abnormally expressed in prostate cancer. As shown in Fig. 2b, compared with normal cells, the expression of miR-338-3p in prostate cancer was significantly downregulated. No significant dysregulation was observed in other screened miRNAs (data not shown). The activity of DSCAM-AS1 luciferase dramatically decreased after transfection of miR-338-3p mimics, but it was significantly enhanced after transfection of miR-338-3p inhibitors (Fig. 2c). As demonstrated in Fig. 2d, miR-338-3p expression increased considerably following DSCA-AS1 knockdown, which was reversed by transfection with the miR-338-3p inhibitor.

### The biological effect of FAM66C/miR-338-3p in prostate cancer

**Fig. 3:**
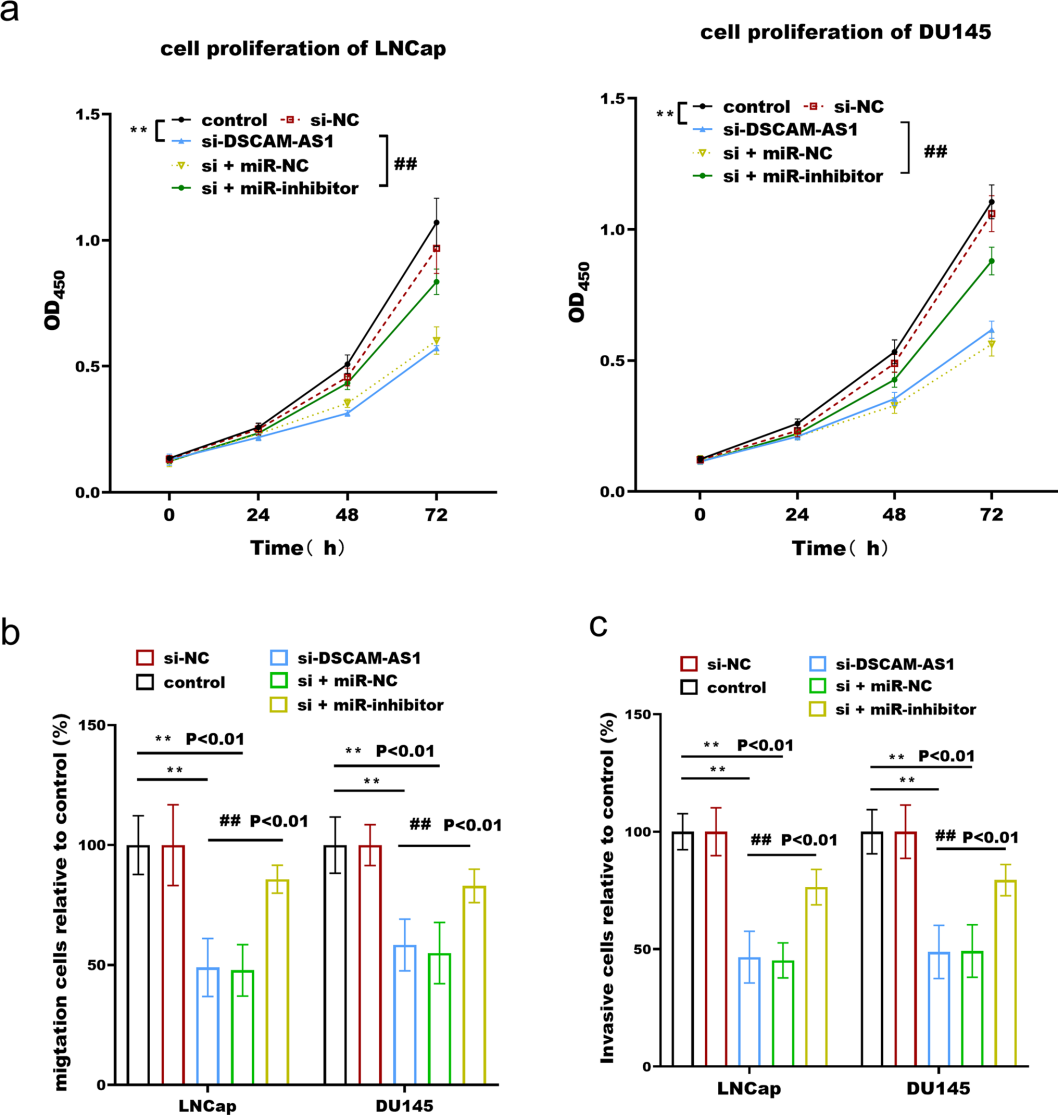
The effect of DSCAM-AS1 / miR-338-3p on cell proliferation (**a**), migration (**b**) and invasion (**c**) of prostate cancer. DSCAM-AS1 promotes the proliferation, migration and invasion of prostate cancer cells, while silencing miR-338-3p reverses the inhibitory effect of DSCAM-AS1 knockout. **p < 0.01, compared with control group; ##*p* < 0.01 compared with the si-DSCAM-AS1 group

Prostate cancer cells proliferated less after DSCAM-AS1 was silenced, however, this result was reversed following miR-338-3p inhibitor transfection (Fig. 3a). The cell migration and invasion of prostate cancer were also decreased by DSCAM-AS1 knockdown (Fig. 3b, c), and the inhibitory effect was also reversed by transfection with the miR-338-3p inhibitor.

## Discussion

The increasing morbidity and mortality of prostate cancer are seriously affecting the healthy lives of men [16]. Currently, the 5-year survival rate following treatment for patients with early-stage prostate cancer is very near 100%, while for patients with advanced prostate cancer, it is fewer than 30% [17]. It has been discovered through extensive research on prostate cancer that the abnormal expression of several lncRNAs is associated with various physiological activities of prostate cancer cells and can be exploited as a target for early diagnosis and prognosis of prostate cancer [18].

DSCAM-AS1 is closely linked to the development of certain malignancies, and its upregulation is linked to carcinogenic activities, which portends a deterioration in the prognosis of patients [19]. In osteosarcoma, the expression of DSCAM-AS1 was upregulated, which can be a warning sign of poor prognosis [20]. Wang et al. found that tumor protein D52 (TPD52) is closely related to the development of hemangiomas, and in hemangiomas, TPD52 is miR-411-5p is a downstream target, whereas DSCAM-AS1 can act as a competing endogenous RNA (ceRNA) to regulate miR-411-5p, thereby regulating the progress of hemangioma [21]. DSCAM-AS1 expression is associated with poor prognosis in patients with colorectal cancer. Silencing DSCAM-AS1 can inhibit the proliferation and migration of colorectal cancer cells [22]. In endometrial adenocarcinoma, DSCAM-AS1 promotes tumor transcription and has carcinogenic effects [23]. This is consistent with the results of this study, which further demonstrates the reliability and science of this study. In the tissues and cells of prostate cancer, the expression of DSCA-AS1 was dramatically elevated. In the clinical symptoms of prostate cancer patients, tumor size, AJCC stage, lymph node metastasis, Gleason score, and PSA, which indicated the progression and severity of the tumor, were all correlated with the expression of DSCAM-AS1. It was shown that the high expression of DSCAM-AS1 can be a sign of malignancy. DSCAM-AS1, AJCC stage, differentiation, lymph node metastasis, and PSA have the potential to serve as prognostic bioindicators for prostate cancer patients. Patients with relatively high expression of DSCAM-AS1 have a higher mortality rate. All the above indicates that DSCA-AS1 has the potential to be a therapeutic target for prostate cancer and can be effectively used as a marker for poor prognostic.

Although lncRNAs cannot code protein directly, they can act as competitive endogenous RNAs, targeting miRNAs and disrupting the binding between miRNAs and coding RNAs, so probing the regulatory mechanism between lncRNA/miRNAs provides new and important avenues for cancer treatment [24]. A variety of miRNAs are regulated by DSCAM-AS1, including miR-338-3p [19]. This study found that miR-338-3p is the downstream gene of DSCAM-AS1, which is abnormally expressed in prostate cancer. In cervical cancer, DSCAM-AS1 can act as an oncogene and function by sponging miR-338-3p [25]. Ji et al. found that in liver cancer, the direct target of DSCAM-AS1 is miR-338-3p, which can partially reverse the inhibitory effect of DSCAM-AS1 knockout. By sponging miR-338-3p, DSCAM-AS1 can promote the proliferation of liver cancer cells [26]. DSCAM-AS1 negatively regulates miR-338-3p expression and knocking down DSCAM-AS1 dramatically increases miR-338-3p expression. miR-338-3p inhibitors reversed the inhibitory effect of DSCAM-AS1 knockdown on prostate cancer cell proliferation, migration, and invasion. It has been reported that miR-338-3p is down-regulated and targets RAB23 in prostate cancer and the Rab subfamily is a key regulator of cell membrane traffic. When the expression of miR-338-3p is inhibited, the expression of RAB23 protein will be activated, thus promoting the cell proliferation and metastasis of prostate cancer cells [27]. Therefore, it is speculated that DSCAM-AS1 activates the expression of RAB23 protein through miR-338-3p, thereby enhancing the interaction between cell membrane and the outside world and promoting the development of cancer cells.

We found that AJCC stage, differentiation, lymph node metastasis PSA, and Gleason score were all significantly correlated with DSCAM-AS1, but only AJCC and PSA were found to be predictive markers of prognostic risk factors. It has been reported that age is closely related to prostate cancer, and older people have a higher risk of developing prostate cancer and poor prognosis [28], while this study shows that age is not significantly associated with the prognosis of prostate cancer (p < 0.05). It is speculated that this may be caused by the small sample size, and the sample size will be further expanded in the follow-up experiment for exploration. In addition, we found an average expression of DSCAM-AS1 in four cell lines with different cellular staging, however, the four cells were of different origins, with LNCaP from lymph node metastasis, PC3 from bone metastasis, Du-145 from brain metastasis, and 22RV1 from the non-metastatic portion.Whether this is related to the mechanism of action of DSCAM-AS1 deserves further investigation.

## Conclusion

In conclusion, DSCAM-AS1 can be used as an oncogene to promote the development of prostate cancer by down-regulating miR-338-3p. This opens up a broader prospect for promoting the development of prostate cancer and triggers further thinking on the application of lncRNA in prostate cancer. Nevertheless, the results above are based on cell experiments and a single sample size, and more clinical and in vivo zoological experiments are needed to confirm them.

### Supplementary Information

**Additional file 1: Table S1.** Genetic data set details.

**Additional file 2: Table S2.** RT-qPCR primer sequences.

## Data Availability

All data generated or analysed during this study are included in this article. Further enquiries can be directed to the corresponding author.
