# Supplementary material for: DSCAM-AS1 promotes the development of prostate cancer
Source: Discov Oncol. 2024 Apr 11;15:113. doi: 10.1007/s12672-024-00931-3 (PMC11009176; doi:10.1007/s12672-024-00931-3)
Supplement: Supplementary file 1 — Additional file 1: Table S1. Genetic data set details. [file 12672_2024_931_MOESM1_ESM.docx]

Table S1 Genetic data set details

| **Data set** | **Chip type** | **platform** | **Number of prostate tumor tissue samples** | **Number of normal prostate tissue samples** |
| --- | --- | --- | --- | --- |
| GSE179321 | Microarray chip | GPL24676 | 3 | 3 |
| GSE115414 | Microarray chip | GPL16791 | 16 | 8 |
| GSE229904 | Microarray chip | GPL18573 | 236 | 35 |
