# Supplementary material for: DSCAM-AS1 promotes the development of prostate cancer
Source: Discov Oncol. 2024 Apr 11;15:113. doi: 10.1007/s12672-024-00931-3 (PMC11009176; doi:10.1007/s12672-024-00931-3)
Supplement: Supplementary file 2 — Additional file 2: Table S2. RT-qPCR primer sequences. [file 12672_2024_931_MOESM2_ESM.docx]

Table S2 RT-qPCR primer sequences

| Primer name | | Primer sequence |
| --- | --- | --- |
| DSCAM-AS1 | forward | 5'-ACAGAGATGGACGACGGATC-3' |
|  | reverse | 5'-TTGTGAGCCTGAGAGATCCC-3' |
| GAPDH | forward | 5'-GGAGCGAGATCCCTCCAAAAT-3' |
|  | reverse | 5'-GGCTGTTGTCATACTTCTCATGG-3' |
| miR-338-3p | forward | 5'-TGCGGTCCAGCATCAGTGAT-3' |
|  | reverse | 5'-CCAGTGCAGGGTCCGAGT-3' |
| U6 | forward | 5'-TTCGGCAGCACATATAAATTGG-3' |
|  | reverse | 5'-CGCTTCAATTTGCGTGTCAT-3' |
